# Correlates of resilience among university students in Oman: a cross-sectional study

**DOI:** 10.1186/s40359-022-01035-9

**Published:** 2023-01-05

**Authors:** Omar Al Omari, Asma Al Yahyaei, Dianne Wynaden, Jalal Damra, Maen Aljezawi, Mohammad Al Qaderi, Huda Al Ruqaishi, Loai Abu Shahrour, Mohammed ALBashtawy

**Affiliations:** 1 College of Nursing, Sultan Qaboos University, P.O. Box 66, Al-Khoudh, 123 Muscat, Oman; 2 School of Nursing, Curtin University, Perth, WA Australia; 3 Educational Psychology and Counseling Department, Hashemite University, Zarqa, Jordan; 4 Psychology Department, Sultan Qaboos University, Muscat, Oman; 5 Princess Salma Faculty of Nursing, AL al-Bayt University, Mafraq, Jordan; 6 Sultan Qaboo University Hospital, Muscat, Oman; 7 Nursing Department, Higher Colleges of Technology, Ras Al Khaimah, UAE

**Keywords:** Resilience, University students, Well-being, Self-esteem, Physical health

## Abstract

**Background:**

Resilience has a paramount role in dealing with different life stressors and has a great impact on mental health.

**Aim:**

To assess the level of resilience among university students and explore the relation between resilience and a number of variables including psychological well-being, self-esteem and physical health.

**Methods:**

A cross-sectional design was utilized. Data was collected from 676 university students. Students were required to complete a demographic sheet, Brief Resilience Scale, World Health Organization Well-Being Index, Rosenberg Self-Esteem Scale and a physical health survey.

**Results:**

Overall, less than half of the participants have reported low levels of resilience (45.3%; n = 306). Regular sleep, perceived stress, WHO well-being index, self-esteem, and having a cumulative GPA of more than two, were factors that significantly predicted positive resilience in multivariate analysis.

**Conclusion:**

Resilience is a necessary skill among university students that requires more academic attention. Factors predicting positive resilience should be considered when implementing mental health promotion programs.

## Introduction

The complexities and fast-paced rhythm of contemporary life, and the consequent social and economic stressors, make a mental health assessment paramount [1]. University students, as a part of society, inevitably have the same stressors, in addition to stressors that result from the transition to university life, like financial demand, study workload, and being accepted by peers [2]. This can have a profound effect on their academic achievement [3–5], psychological well-being [6–8], and physical health [9–12]. The cumulative effect of these stressors can make the students more vulnerable to poor psychological well-being, especially since most of them come to the university without any previous preparation or education on how to adapt and manage these stressors [13]. For example, a recent study reported the total prevalence of stress, anxiety, and depression, was 31%, 39.4%, and 56.1%, respectively among Omani university students [14]. In line with this Jahan et al. [15], Al-Busaidi et al. [16]. The psychological well-being of young adults, such as university students, has been adversely correlated with psychological distress and inefficient coping mechanisms [17]. As a reaction, researchers explored “resilience” as an is important factor that help students bounce back. Research has shown the role of resilience as a framework that explains why some students adapt to the university life and others do not [18, 19]. Having said that, resilience is not solely responsible for adaptation—other psychosocial factors including peer pressure, affiliation to the university, and presence of social support also have a role.

Although previous studies reported different definitions of resilience, all of them agreed that the resilience of a person is a dynamic adaptive process to manage stress and return to the normal state [3, 13, 20–22]. Resilient students are better equipped to adjust to and handle the rigors of university life and prevent psychological stress [23]. Different features of resilience, such as confidence, optimism, will to challenge, having a meaningful objective and perseverance, have a negative relationship with anxiety and stress events [24, 25]. Resilience is also linked with high levels of religion and spiritual practice [26], life satisfaction [27], self-esteem and decision-making [28], and coping mechanisms [29].

Literature reported different levels of resilience among university students, along with factors correlating with it. In general, most of the published studies reported moderate resilience among undergraduate students [30–32]. For instance, a study conducted in Jordan found a moderate level of resilience among university students, with resilience being negatively correlated with depression and positively with social support from family or friends [31]. Other studies also found moderate level but with different correlates of resilience—psychological well-being, optimism, and mindfulness [33]. Also, number of studies only explored predictors of resilience, without measuring its level. In a recent systematic review, it was found that psychological well-being is the most reported predictor of resilience [22]. Different studies found quality of life [34], physical health, and supportive parenting to be correlated with resilience [17]. Other researchers explored the relation between academic achievement and resilience. In this domain, one study found that resilience predicted academic achievement, meaning that the higher the level of resilience, the better academic achievement was [17, 35]. In the Sultanate of Oman, where current study was conducted, only one study explored the relationship between resilience and psychological wellbeing among a group of university students, and found a positive link between mental health and resilience [36].

Despite the importance of resilience and its correlation to stress and mental health, physical health and academic achievement, it is still under study among university students. Different studies used different scales to measure resilience, physical and psychological well-being, and were also conducted in different cultures with different perceptions of psychosocial support and quality of life, which makes it difficult to generalize which are the factors that most correlate with resilience [21]. There is a need for more studies in the Omani context to validate the findings of the previous study, because Oman has cultural aspects and patterns of social support that differ from the other countries where similar studies were conducted. The aim of the current study was to (1) describe some characteristics of Omani university students; (2) assess the level of resilience; (3) explore the relationship between resilience among Omani university students and selected university students’ characteristics; (4) explore the relationship between resilience, number of hours’ students sleeps every night; number of hours mobile phone is used every day, perceived stress, psychological well-being, self-esteem, and physical health; (5) identify the extent to which some variables, including demographics, perceived stress, psychological well-being, self-esteem, and physical health, could be correlated with resilience using multiple linear regression. A significant relationship between resilience and the aforementioned factors is expected. Identifying these factors and including them in future intervention programs that targeting university students can improve overall university students’ physical and mental health, and decrease the possibility of anxiety and depression, eventually contributing to higher degrees of academic achievement.

## Methods

### Design

The study adopted a descriptive cross-sectional design, utilizing a self-administered questionnaire survey circulated among Omani students at Sultan Qaboos university. The data collection took place between the October and December 2021.

### Participants

Students from a university were gathered using a convenience sample technique. The students that took part were all required to meet the following requirements: (a) they had to be enrolled for the current semester; (b) they had to be able to read and understand English; and (c) they had to be able to give informed consent. The data did not include anyone who were taking exams at that time. Sultan Qaboos University provided the data for this study. It is the sole state university in Oman, and students from all over the country are admitted to the university.

The minimum sample size was calculated using Slovin’s method (n = N / (1 + N e^2^)), where n is the number of participants, N is the total population, and e is the margin of error (0.05). Since there are 16,000 students at the Sultan Qaboos University, 400 university students were required as minimum sample size.

### Data collection procedure

Data was gathered through self-reported questionnaires. The survey and a packet with details about the study’s intent and consent form were provided to students by their colleges.

The research packages were given to the deans’ assistants in each college affiliated to the university. They were given full details about the study and had been asked for their assistance in distributing the questionnaire, and all were happy to cooperate. On behalf of the primary investigator, the deans’ assistants then asked the students to participate, and if they agreed, they were given the research package. Participants were given ten days to return the questionnaire completed in a sealed envelope and put it anonymously in a designated box located in the college lobby, to be collected later by the primary investigator. The total number of the questionnaires distributed was 1000, to achieve a greater number of responses than the calculated sample size. The questionnaire took approximately ten minutes to be completed.

### Survey

The self-administered questionnaire consisted of five parts: demographics, Brief Resilience Scale (BRS), World Health Organization Well-Being Index (WHO-5), and Physical Health Status, Rosenberg Self-Esteem Scale (RSES). The score from the BRS represents the resilience level, which is the outcome variable (dependent variable). The other four parts represent the independent variables that were hypothesized to affect the outcome variable. Below are the details of these parts.

### Demographics

This section of the questionnaire was intended to collect data related to students’ demographics. These were: age, gender, current marital status, place of residence, academic year, GPA, cumulative hours, work status, father’s educational level, mother’s educational level, monthly family income, number of family members, student’s health in general, daily sleeping hours, and presence of chronic illness. In this regard, the researchers identified the meaning of chronic illnesses and provide some examples of the most popular chronic illnesses. Then the participants were asked to self-report whether they have a chronic illness or not.

#### Brief resilience scale (BRS)

This six-items scale assesses resilience (i.e., the ability to bounce back under stressful situations) [37]. “I tend to bounce back quickly after hard times” is an example of items used. The scale goes from 1 (Strongly Disagree) to 5 (Strongly Agree). It has been cross-culturally validated and found to be reliable in a number of populations (Cronbach’s alpha ranges from 0.80 to 0.90). The survey is available in public domain and free to use.

#### World Health Organization well-being index (WHO-5)

This is a common scale for assessing psychological well-being [38]. The scale includes items on depression (negatively phrased, ‘I have felt cheerful in good spirits’), fatigue (negatively phrased, ‘I woke up feeling fresh and rested’), and anxiety and stress (negatively phrased, ‘I have felt calm and relaxed’). The scale has been used to assess clinical depression as a proxy measure. The responses ranged from 0 (never) to 5 (always). The final score was calculated by multiplying the raw score by four. Individuals who receive a score of 50 or lower will be considered as having poor wellbeing. The scale has been validated in clinical and general populations across cultures, with consistent reliability (Cronbach’s alpha range from 0.88 to 0.93) [39]. This survey too is in the public domain and can be used for free.

#### Physical health status

The participants’ self-reported physical health state was investigated using a single item question, ‘How do you assess your current physical health status?’ Responses range from ‘Poor’ to ‘Excellent.’

#### Rosenberg self-esteem scale (RSES)

The 10-item Rosenberg Self-Esteem Scale (RSES) was developed by Rosenberg in 1965 to assess self-esteem [40]. All questions are answered on a 4-point Likert scale, from strongly agree to strongly disagree. A score of less than 30 shows a low self-esteem [41]. The Cronbach’s alpha coefficient for the survey was 0.86 [41]. The survey is available in public domain and free to use.

### Ethical consideration

Before the data collection started, ethical approval was obtained from the research and ethics committee at the university (CON/DF/2021/1). The Helsinki Declaration (1989) was used as a guide for our research study. Informed consent procedures were carried out prior to participation to ensure that everyone who was a part of the study was able to make an informed decision to participate on their own volition. Participation was voluntary and participants had the right to withdraw at any time and decline to answer any questions.

### Data analysis plan

SPSS program version 23 was used to analyse the data. Sample demographics were established using descriptive statistics. Independent sample t-tests and one-way analyses of variance (ANOVA) were used. A Pearson correlation was used to test the relationship between the continuous variables. The significant associated factors with resilience were identified using a linear regression model. In all analyses, the significance level was maintained at *p* < 0.05.

## Results

Out of the 1000 surveys distributed, 676 were answered, yielding a response rate of 67.6%. The mean age of the students were 20.77 years and seven in ten of the participants were female (n = 468). A fifth of the participants were from the college of nursing (n = 134) and under a third have a cGPA between 2.75 and 3.29. Most of the participants were single (93.3%, n = 631), and reported the absence of any chronic illness (n = 613). The students reported an average of six hours of nightly sleep (SD = 1.277) and 5.6 h of mobile phone usage (SD = 2.69). About 45.3% (n = 306) of the students had a low resilience level, almost half of them students have a normal resilience level (n = 333), and only 5.5% (n = 37) reported a high resilience rate (see Table 1).

**Table 1:** Participants’ characteristics

Variables	M	SD	Variables	M	SD
AGE	20.9	2.50	PSS	9.3	3.3
BRS	2.99	0.78	WHO-5	46	18.6
RSES	28	6.4	Credit hours completed	50.1	39.4
Number of hours sleep	6.4	1.23	Smartphones use every day	5.7	2.70

Variables	N	(%)	Variables	N	(%)
*Gender*			*Marital status*		
Male	208	30.8	Married	45	6.7
Female	468	69.2	Single	631	93.3
*Self-report of chronic illness*			*Place of living*		
Yes	63	9.3	On campus	331	49.0
No	613	90.7	Off campus	345	51.0
*BRS*			*I live currently*		
Low resilience	306	45.3	Alone	81	12
Normal resilience	333	49.3	With Family	306	45.3
High resilience	37	5.5	With Friend	287	42.5
*College of*			*cGPA*		
Agricultural	50	7.4	< 2.0	41	6.1
Arts	79	11.7	2.001–2.29	76	11.2
Education	59	8.7	2.30–2.74	174	25.7
Engineering	58	8.6	2.75–3.29	204	30.2
Law	51	7.5	3.30–3.74	110	16.3
Nursing	134	19.8	3.75–4.00	26	3.8
Medicine	53	7.8	*Income*		
Science	79	11.7	< 1000	283	41.9
Economics	71	10.5	1001–1500	181	26.8
			1501–2000	98	14.5
			> 2000	114	16.9

*RSES* Rosenberg self-esteem scale, *PSS* perceived stress scale, *WHO-5* World Health Organization Well-Being Index, *BRS* brief resilience scale

The difference in mean between resilience and variables (consisted of three levels and more including cGPA, income, and college type) was investigated using ANOVA test. Findings show that there is a significant difference in mean between resilience and cGPA, college type, and with whom students live (*P* < 0.05). A post hoc (Tukey HSD) test shows a significant mean difference in their levels of resilience between students who live alone and those who live with their friends. There was also a significant difference based on cGPA, between students who scored less than 2 and students who scored 2.75 and more. As expected, there were significance differences in the total mean scores of resilience between students from the college of law on one side and the college of nursing and medicine on the other. An independent t-test was also used to compare the overall mean resilience levels in relation to different dichotomous characteristics. There was no significant difference in the level of resilience across gender, marital status, and history of chronic illness (*P* < 0.05) (see Table 2).

**Table 2:** Bivariate analysis of resilience

Variables	M	SD	P	Cariables	M	SD	P
*Marital status*				*Gender*			
Married	3.2	0.87	0.17	Male	3.1	0.82	0.63
Single	3.0	0.79		Female	3.0	0.79	
*Do you have chronic illness*				*Place of living*			
Yes	2.9	0.70	0.48	On campus	3.0	0.79	0.92
No	2.9	0.80		Off campus	3.0	0.80	
*College of*				*I live currently*			
Agricultural	3.0	0.89	**< 0.01**	Alone	2.8	0.83	**0.01**
Arts	2.9	0.84		with Family	2.9	0.81	
Education	3.1	0.81		with Friend	3.0	0.76	
Engineering	2.8	0.79		*cGPA*			**0.01**
Law	2.7	0.86		< 2.0	2.6	0.95	
Nursing	3.1	0.56		2.001–2.29	2.9	0.82	
Medicine	3.2	0.76		2.30–2.74	2.9	0.79	
Science	2.9	0.83		2.75–3.29	3	0.78	
Economics	2.9	0.90		3.30–3.74	3.0	0.79	
*Income*				3.75–4.00	3.3	0.73	
< 1000	2.9	0.75	0.15				
1000–1499	2.9	0.76					
1500–1999	2.8	0.89					
> 2000	2.9	0.78					

A Pearson correlation test shows a significant positive relationship between resilience, hours of sleep every night, Well-Being Index, general physical health and self-esteem. However, a moderately significant negative relationship was detected between resilience and perceived stress (see Table 3).

**Table 3:** The Relationship Between the resilience and selected continuous variables

		1	2	3	4	5	6	7	8	9
1	BRS	1	0.06	0.02	0.08*	− 0.01	− 0.46*	0.36*	0.55*	0.20*
2	Age	0.06	1	0.41*	− 0.09*	− 0.08*	− 0.09*	0.04	0.04	0.02
3	Credit hours completed	0.02	0.41*	1	− 0.09*	− 0.01	− 0.04	− 0.01	− 0.01	0.06
4	Hours’ sleeps every night	0.08*	− 0.09*	− 0.09*	1	0.08*	− 0.10*	0.08*	− 0.01	0.07
5	h mobile phone use/ day	− 0.01	− 0.08*	− 0.01	0.08*	1	0.05	− 0.02	− 0.05	− 0.10*
6	PSS	− 0.46*	− 0.08*	− 0.04	− 0.10*	0.05	1	− 0.56*	− 0.49*	− 0.27*
7	WHO	0.36*	0.05	− 0.013	0.09*	− 0.03	− 0.56*	1	0.34*	0.33*
8	RSES	0.55*	0.04	− 0.01	− 0.01	− 0.05	− 0.49*	0.34*	1	0.25*
9	General physical health	0.20*	0.02	0.07	0.071	− 0.11*	− 0.27*	0.33*	0.25*	1

*Correlation is significant at the 0.05 level; *RSES* Rosenberg self-esteem scale, *PSS* Perceived stress scale, *WHO-5* World Health Organization Well-Being Index, *BRS* Brief resilience scale

The associated factors with resilience were identified using a linear regression model. To evaluate multicollinearity, a correlation matrix was produced first, followed by the testing of other multiple linear regression assumptions. Finally, dummy variables were created from variables with more than two categories. The statistical model was filled in with the independent variables that were significant at the bivariate level. The researchers used stepwise method to exclude variables till they reach to the parsimonious model. The regression model was significant F (9,620) = 42.032, *P* < 0.05, R^2^ = 0.38 compared with the constant. Overall, resilience was significantly associated with regular sleep, perceived stress, WHO well-being index, self-esteem, and having a cumulative GPA more than 2. See Table 4.

**Table 4:** Associated factors with resilience

Model	Unstandardized coefficients		Standardized coefficients	t	Sig.	95.0% confidence interval for B	
	B	SE	*β*			Lower Bound	Upper Bound
(Constant)	1.009	0.282		3.573	< 0.01	0.454	1.563
Sleep every night	0.041	0.021	0.064	1.990	0.047	0.001	0.082
PSS	− 0.041	0.010	− 0.168	-4.079	0.000	− 0.061	− 0.021
WHO-5	0.005	0.002	0.107	2.781	0.006	0.001	0.008
RSES	0.055	0.005	0.444	12.004	< 0.01	0.046	0.064
cGPA (relative to < 2.0)							
2.001–2.29	0.413	0.124	0.168	3.334	0.001	0.170	0.657
2.30–2.74	0.420	0.111	0.234	3.774	0.000	0.201	0.638
2.75–3.29	0.365	0.110	0.214	3.332	0.001	0.150	0.581
3.30–3.74	0.327	0.117	0.154	2.782	0.006	0.096	0.557
3.75–4.00	0.474	0.160	0.118	2.959	0.003	0.160	0.789
Model R^2^	0.38						

*RSES* Rosenberg self-esteem scale, *PSS* Perceived stress scale, *WHO-5* World Health Organization Well-Being Index, *BRS* Brief resilience scale

## Discussion

The main purpose of this study was to assess the level of resilience. In the current study, 45.3% reported low levels of resilience, 49.3% normal resilience, and 5.5% high resilience level. This finding is consistent with the previous study in the region, in which 50% university student reported normal to high level of residence, (29) and differs from what was reported by Johnson (40), who found a higher level of resilience among university students in western societies [42]. There is a need to improve resilience level among university students in the Arabic country to match their counterparts in the western countries. In order to do so, it is important to explore factors associated with resilience, which is the second objective of the current study.

Resilience in the current study was significantly associated with sleeping every night, perceived stress, WHO well-being index, self-esteem, and having a cumulative GPA of more than 2.

Worldwide, insufficient sleep is a major concern among the university student population, which could impact their overall health negatively [43]. The result of this study has shown that duration of sleep significantly associated with the students’ level of resilience. These findings are in line with those of other studies that suggest that sleep and resilience are positively correlated [21, 43]. However, the emphasis of the published literature was more on the quantity of sleep rather than the quality [21]. Thus, future research is required to validate the direction of this association through carefully designed studies that examine the effect of quality of sleep on the level of resilience.

Moreover, the multivariable regression analysis on the level of resilience indicated that more hours of sleep at night, lower perceived stress as measured by PSS, increased level of well-being as measured by the WHO-5, and higher cGPA were associated with higher reporting of resilience. These results are similar to those of prior studies that have investigated the relation between the wellbeing and level of resilience [13, 44]. However, the relationship between these associated factors and resilience is thought to be bidirectional. This means that it cannot be determined whether high resilience promoted the previously mentioned factors, or whether the presence of these factors lead to high resilience among students. Longitudinal studies are required to determine the direction of the relationship.

Additionally, consistent with the literature, this research found that higher self-esteem level among university students was significantly associated with high levels of resilience [2, 45]. In fact, the relationship between self-esteem and resilience is reciprocal in nature where having a high level of self-esteem will enhance the person’s ability to endeavour and bounce back during difficult times to maintain a high level of resilience. On the other hand, if an individual is naturally resilient, they will also have high self-esteem due to effective coping mechanisms against stress.

In term of academic performance, particularly a GPA higher than 2 was identified as positive predictor of the students’ resilience. Although these results differ from some published studies which were not able to detect any association between the students’ resilience and the students’ GPA [1], they are consistent with those of which identified a significant association between academic performance and resilience. Some authors have justified this correlation by arguing that a higher cGPA leads to academic satisfaction and self-efficacy, which improve self-confidence among students [46, 47]. This result may also be explained by the fact that student with higher grade would be able to overcome different type of challenges and stressor by using healthy adaptive strategies which eventually enhance their resilience level.

On the other hand, this study identified the students’ level of perceived stress as the negative associated factor with resilience. In contrast to these findings, Alatawi and Morsy [20] have revealed no significant correlation between resilience and stress. However, the results of this study corroborate the findings of previous publications which have reported a negative correlation between resilience and stress [48–51]. In fact, some previous studies identified resilience as a protective factor against stress, since they have defined personal resilience as an individual’s mental capability to resist and cope against significant stress, hardships and trauma.

Although the study has successfully generated new knowledge about the factors associated with resilience among university students in Oman, it has certain limitations in terms of method and design. First, our findings cannot explain the causal links between resilience and independent variables due to the limitations of a cross-sectional methodology. That is, though the researchers were aware to the theoretical models consider resilience as a predictor of psychological well-being, the researchers, from a statistical point of view, used resilience as an outcome variable in the regression model to explore the relationship between it and each variable after controlling the reset of variables. Therefore, readers should read the resilience and the rest of the significant variables as associated factors and not in the context of predictors and outcomes. Future research should focus on doing prospective longitudinal or cohort studies to determine the temporal sequence of the variables. One of the main drawbacks of this study was utilizing the questionnaire as a data collection method, which did not offer the researcher an opportunity to follow up ideas and clarify issues.

In conclusion, although resilience is multifaceted and multidimensional concept in nature, in this study it was considered as a dynamic, context-dependent adaptation mechanism, which could be enhanced and learned. The knowledge produced by this study could be used to develop strategies to reinforce the students’ resilience skills to prepare them for facing and overcoming their daily challenges. It is highly recommended to integrate the resilience skills into the existing curricula at the national and SQU level. Establishing courses to teach stress management, problem-solving skills, emotional regulation, and coping strategies will enhance students’ resilience. The students’ counselling centre at SQU can also participate by providing more focused workshops about the aforementioned topics and direct one-to-one counselling may also help. These experiences can be shared with and transferred to other higher education institutions at the national level. Another recommendation for educators is to involve the students in self-care programs where they will learn how to take care of themselves physically and mentally, which will allow them to eventually be able to think more clearly, enabling them to be better equipped to handle challenges.

## Data Availability

The datasets used and/or analyzed during the current study are available from the corresponding author on reasonable request.
